# Internal and marginal fits of 3D-printed provisional prostheses: comparative effect of different printing parameters

**DOI:** 10.3389/froh.2024.1491984

**Published:** 2024-12-12

**Authors:** Yousif A. Al-Dulaijan, Rand Aldamanhori, Hadeel Algaoud, Rand Alshubaili, Reem Alkhateeb, Haidar Alalawi, Reem Abualsaud, Firas K. Alqarawi, Faisal D. Al-Qarni, Mohammed M. Gad

**Affiliations:** ^1^ Department of Substitutive Dental Sciences, College of Dentistry, Imam Abdulrahman Bin Faisal University, Dammam, Saudi Arabia; ^2^ College of Dentistry, Imam Abdulrahman Bin Faisal University, Dammam, Saudi Arabia; ^3^ Fellowship in Orthodontics Program, College of Dentistry, Imam Abdulrahman Bin Faisal University, Dammam, Saudi Arabia

**Keywords:** 3D printing, provisional restorations, fixed dental prostheses, accuracy, printing parameters

## Abstract

**Objectives:**

The influence of printing parameters on the marginal and internal fit of three-dimensional (3D) printed interim fixed partial dentures (IFPDs) has been understudied. This investigation sought to elucidate the impact of printing orientation and post-curing time on these critical factors.

**Methods:**

A total of 260 3-Unit IFDPs were printed using two different resins (130/NextDent C&B MFH and 130/ASIGA DentaTOOTH). For each material, specimens were printed with three different angulations (0-, 45-, and 90-degree in relation to the z-axis). Each was further divided into 4 groups (*n* = 10) according to post-curing time (30-, 60-, 90-, and 120 min), while the green state (GS) group at 0-degree remained without post-curing as a control. Each specimen was scanned and then superimposed on the original CAD file. The marginal and internal fit of premolar and molar restorations were evaluated using the silicone replica technique. Digital scanning of the master die, both with and without a fit checker, was followed by data superimposition to compare the master die with the fit checker of each sample. 3D comparisons were conducted using initial and best-fit alignment methods, and the root mean square error (RMS) was calculated to quantify marginal and internal fit at each abutment and for the overall restoration. Statistical analysis was performed using JMP® software (JMP®, Version 16, SAS Institute Inc., Cary, NC, USA, 1989–2022) with a significance level 0.05 for all tests.

**Results:**

For the ASIGA group, 0-degree orientation generally exhibited better fit than 45- and 90-degree orientations, with some variations based on post-cure time. For marginal fit, ASIGA crowns typically showed better results with 90-degree orientation, while NextDent crowns demonstrated consistent performance across orientations. Post-curing time also influenced marginal fit, with longer durations generally resulting in improved outcomes.

**Conclusion:**

With different printing orientations and post-curing times, ASIGA and NextDent resins can produce IFDPs with acceptable internal and marginal fit. However, NextDent resin consistently outperformed ASIGA in terms of overall fit. Further research is needed to evaluate the long-term clinical performance of these materials.

## 1 Introduction

The fabrication of provisional restorations constitutes a critical stage in the sequence of fixed prosthodontic treatment (1). Provisional restorations serve as interim replacements for lost tooth structure, safeguarding the prepared tooth biologically and mechanically, ensuring positional stability, and facilitating soft tissue healing and aesthetic maintenance (2, 3). Additionally, they play a pivotal role in addressing aesthetic concerns, verifying occlusion, and evaluating speech before delivering the definitive prosthetic restoration (4).

Marginal integrity, internal fit, and fracture toughness are crucial properties to have in a crown in order to ensure long-term success (5–7). Achieving an optimal marginal fit comparable to that of a definitive prosthesis is paramount for preserving periodontal health and minimizing cement degradation (8). Simultaneously, optimal internal fit ensures an adequate and uniform cement space without compromising the retentive and resistant features of the restoration at the time of cementation (6, 9, 10). Poor internal and marginal adaptation can form a gap, creating space for plaque retention and microbial proliferation (2). This may lead to the dissolution of the luting agent and eventually recurrent caries, as well as periodontal or pulpal disease (11).

Direct fabrication of polymethyl methacrylate (PMMA) provisional restorations in the oral cavity presents several limitations (4), including polymerization shrinkage, which can result in dimensional discrepancies within the restoration and an exothermic reaction that may induce thermal trauma to the pulp (12). In contrast, the indirect fabrication method, involving the creation of a cast from a patient's prepared teeth, mitigates these risks. However, indirect fabrication is susceptible to variations in technician skill and may lack reproducibility (1). Recent methods of IFDP fabrication have been developed through digital technology, which has many advantages over conventional methods (1, 12). Two CAD-CAM fabrication methods are recognized: subtractive (SM) and additive (AM). AM (built as a layer-by-layer) applications in the dental field are increasing due to the advantages over SM. AM is more economical due to the low material waste (unused resin recycling). In addition, it has high reproducibility of varying objects with complex configurations and the ability to produce large quantities simultaneously (13–15). Stereolithography (SLA) and digital light processing (DLP) are the two most prevalent additive manufacturing (AM) techniques employed in dentistry (13). SLA involves the layer-by-layer polymerization of a liquid resin using an ultraviolet laser, making it the most widely utilized method in this field (16–18). DLP, on the other hand, activates light-sensitive monomers through laser projection. A digital micromirror device (DMD) precisely directs the laser beam, enabling the creation of intricate three-dimensional (3D) structures (19–21). The IFDPs fabricated digitally displayed better marginal and internal fit and greater fracture resistance compared to the conventionally produced IFDPs (17). For 3D-printed resin prostheses, a difference in fit was reported depending on the printing conditions. However, when the conditions were optimized, a clinically acceptable fit was obtained (22). Son et al. (23) evaluated the accuracy of interim dental crowns fabricated using 3D-printing and milling and concluded that 3D-printing showed superior accuracy compared to milling. Contrary to the reported findings of better fit of digitally fabricated prostheses over the conventional ones, Wu et al. (3) compared the internal fit and marginal discrepancy of interim crowns fabricated conventionally, 3D-printed, and CAD-CAM milled. They reported that conventionally fabricated crowns showed superior internal fit and lower marginal discrepancy.

Printing parameters, including orientation, layer thickness, and post-curing time (PCT), significantly influence the properties of printed objects (24). Previous research has demonstrated that printing orientation affects the fit and accuracy of 3D-printed interim crowns (25) and IFPDs (2). Yang et al. (2) recommended a 45-degree orientation for optimal results. Jang et al. (22) concluded that a layer thickness of 50 µm combined with 45- or 60-degree printing orientations constitutes the ideal printing conditions for achieving optimal marginal adaptation and internal fit. Regarding the impact of printing orientation on crown fit, Ryu et al. (25) reported that the marginal and internal fit of DLP 3D-printed interim crowns are influenced by the build angle, with 150- and 180-degree orientations demonstrating superior outcomes.

Printed resins require supplementary polymerization processes to enhance monomer conversion and minimize residual content (26). Previous research has indicated that PCT significantly influences the strength of printed objects (24). Studies have demonstrated that post-curing durations of 60–90 min can lead to increased strength (26). Additionally, investigations have shown that extending the PCT (15–120 min) for 3D-printed resins can improve their flexural properties, Vickers hardness, and biocompatibility (27–29).

While prior studies have explored the accuracy and fit of 3D-printed resin prostheses, the influence of printing orientations and PCT on the marginal and internal fit of SLA and DLP 3D-printed IFPDs remains understudied. The null hypothesis for this investigation posits that printing orientations and PCT do not significantly affect the marginal and internal fit of three-unit IFPDs.

## 2 Materials and methods

### 2.1 Sample size calculation

The sample size for this study was calculated based on the findings of Son et al. (23) and Ryu et al. (25), with a desired error margin of 5% and a study power of 80%. Utilizing a sample size calculation method (30) and comparing means, it was determined that 10 specimens per group were adequate. As a result, 260 specimens (130 per resin, *n* = 10/group) were created using the two different 3D-printed resins.

### 2.2 Specimen designing and printing

**Figure 1:**
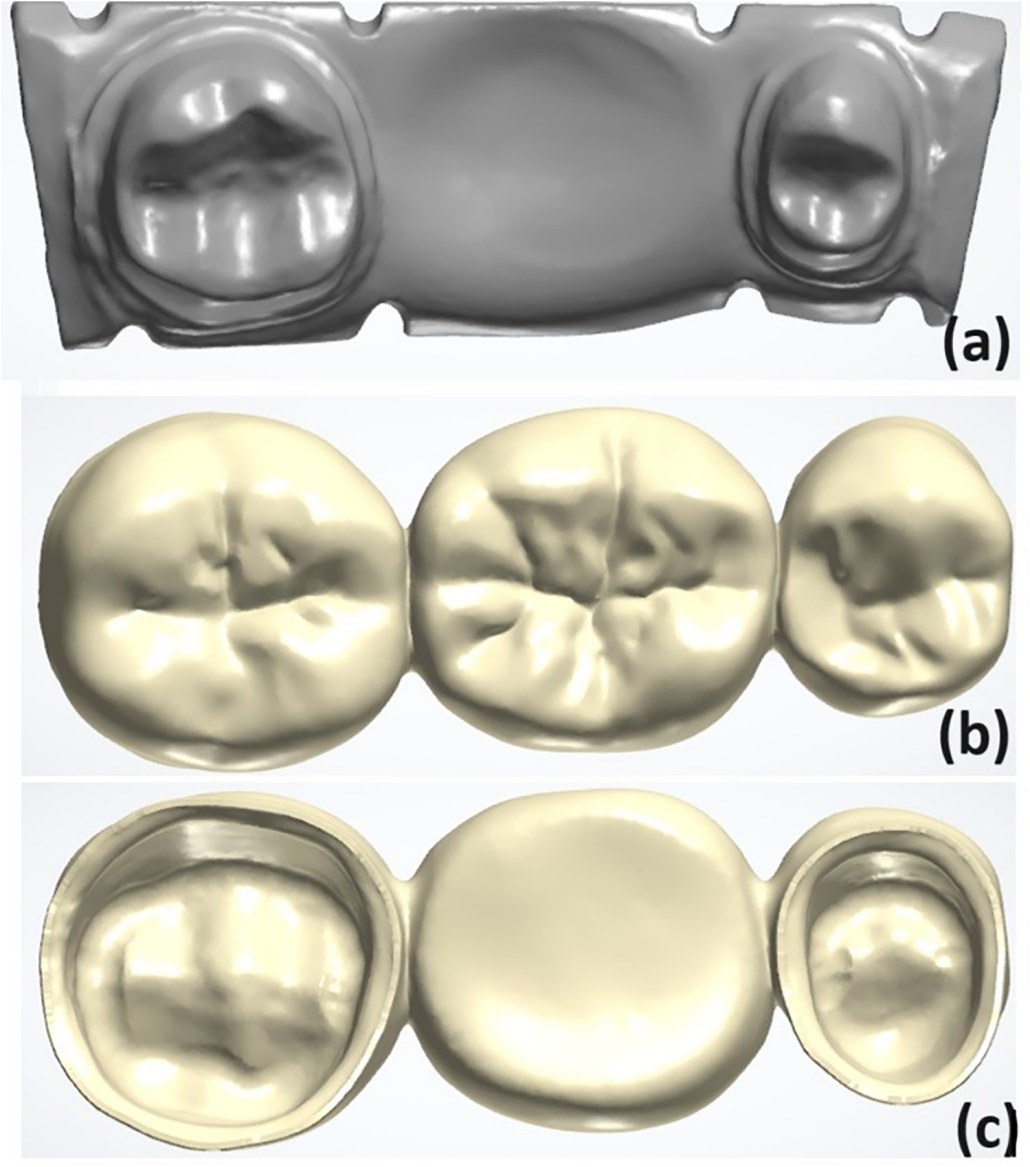
**(a)** Scanned model with reference areas defined. **(b,c)** The designed fixed partial denture (FPD) specimen.

A typodont teeth (right mandibular second premolar and second molar) were prepared to receive 3-unit FPD. The preparation design included a chamfer finish line with a 1 mm axial reduction, a 1.5 mm anatomic occlusal reduction, and a 1.5 mm total occlusal convergence. Four reference points on each side of the base of the prepared model were created using a diamond bur to facilitate the superimposition. The prepared model was scanned using an intraoral scanner (TRIOS 3, 3shape, Copenhagen, Denmark) and converted to a standard tessellation language (STL) file (Figure 1). Following the recommended settings of the 3Shape Dental Software version 2.23.1.0 (3shape, Copenhagen, Denmark), a 3-unit IFDP was designed with a connector size of 15.05 mm^2^ mesially and 14.07 mm^2^ distally, with a modified-ridge lap pontic design and 30 µm cement space starting 1 mm away from the finish line (Figure 1) (19, 31, 32).

### 2.3 Specimens printing

The STL file of the designed specimen was imported to DLP printers (NextDent 5,100 printer, Nextdent B.V., Netherlands) and (Asiga MAX printer, Asiga, Australia). Printing orientation (angle between build directions and the long abutment axis) was set to be 0-, 45-, and 90-degree (Table 1). Supports were positioned on the lingual smooth surfaces to ensure ease of removal. The specimens were printed with a 50 µm printing layer thickness. The printed specimens were ultrasonically cleaned using isopropyl alcohol 99%, then subjected to one of the different PCTs (30-, 60-, 90-, and 120 min/*n* = 10/group). Post-curing device LC-3DPrint Box (3D Systems Corporation, Rock Hill, South Carolina) and Asiga® Flash Cure Box (Asiga, Australia) were used to post-process the NextDent and ASIGA specimens, respectively. One 0-degree printed group (*n* = 10) had no post-curing process as a control for each material. After complete curing, all supports were cut off using a sharp surgical scalpel blade No.15. A x4 magnification loupe was used to detect any defects in the IFDPs. One investigator did the finishing procedures (19, 32). A lightproof box was used to store the printed IFDP specimens after processing and before scanning and testing (22).

**Table 1:** Printing specifications and parameters per 3D-printed resin.

Material	Specifications/parameters	
ASIGA Asiga DentaTOOTH (ASIGA, Erfurt, Germany)	NextDent C&B (CB) NextDent, Soesterberg, Netherlands	Brand name
Methacrylate-based Microhybrid composite resin	Microfilled hybrid methacrylic acid ester-based resin >60% wt methacrylic oligomer (UDMA, EGDMA), 15%–25% wt HEMA	Composition
ASIGA MAX™	Next Dent 5,100	Printer
LED-based Digital Light Processing (DLP)	Digital Light Processing (DLP)	Printing technology
50 µm	50 µm	layer thickness
0-, 45-, 90-degree	0-, 45-, 90-degree	Printing orientations
Asiga Flash, Wavelength: 405 nm	LC-D Print Box, Wavelength: 405 nm	Post-curing machine
30, 60, 90, and 120 min	30, 60, 90, and 120 min	Post-curing time
60°C	60°C	Post-curing Temperature

### 2.4 Fit measurements

Master dies of premolar and molar teeth were initially scanned using a 3Shape TRIOS 3 scanner (3Shape A/S, Copenhagen, Denmark), and the obtained file was designated as the “reference scan”. A thin layer of polyvinyl siloxane impression adhesive was applied to the occlusal surfaces of the prepared teeth. The intaglio surfaces of each FPD were subsequently sprayed with a lubricant layer and filled with fit checker material (FIT CHECKERTM ADVANCED BLUE, GC America Inc., IL, USA). A load of 49.05 N was applied to each IFDP until the material was set. Excess fit checker material was detached using a new #15 blade. A single investigator conducted all procedures to ensure consistency. The use of a lubricant layer ensured the sticking of the fit checker material to the master die.

**Figure 2:**
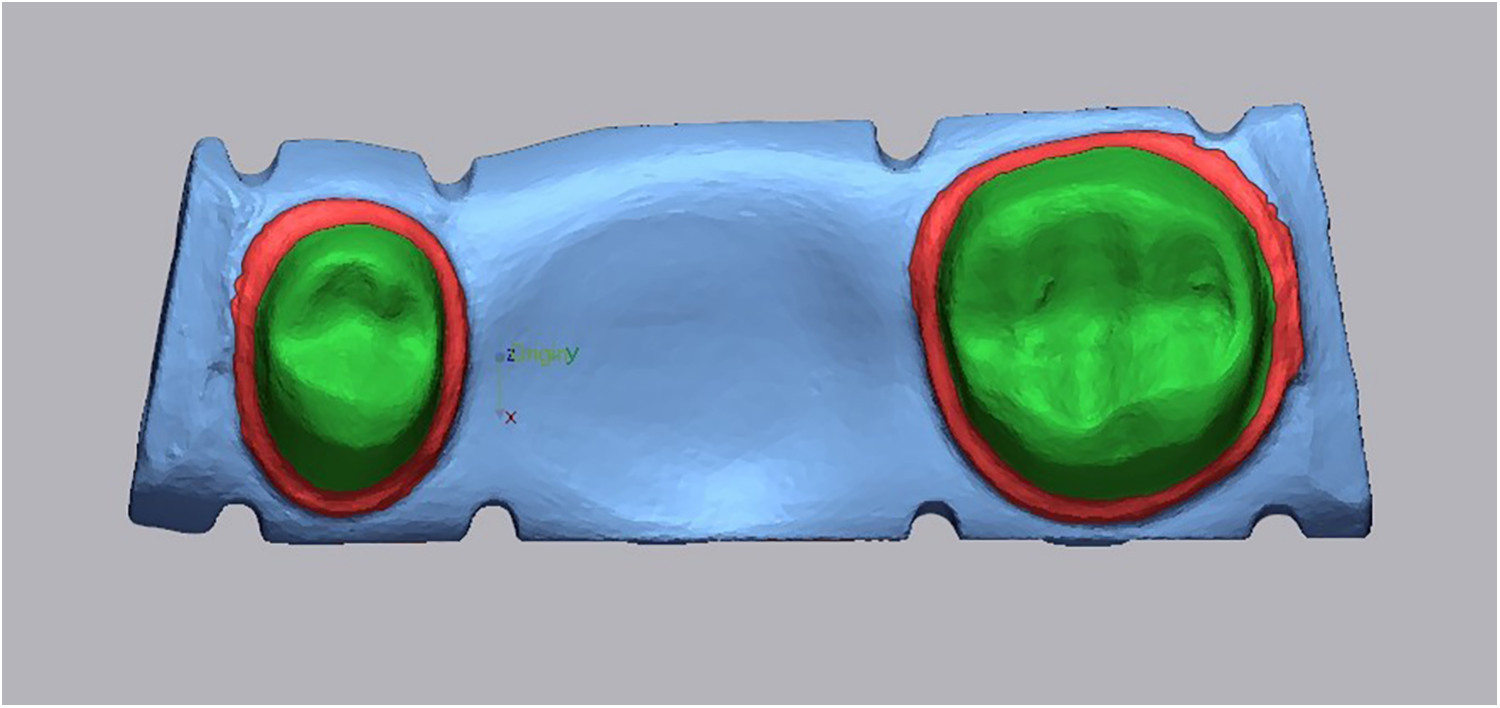
Representative color maps illustrate the comparison areas for marginal adaptation (red) and internal fit (green).

Following, the master die with fit checker material was subsequently rescanned using the same 3Shape TRIOS 3 scanner. The new file was labeled as the “fit scan”. Both the “fit scan” and “reference scan” STL files were superimposed on each other using Geomagic Control X software (3D Systems Inc., SC, USA) for further analysis. The “reference scan” was separated into three areas: internal, marginal, and overall (combining the internal and marginal areas) for each individual abutment (Figure 2).

The “initial alignment” and “best-fit alignment” between the “reference scan” and “fit scan” were performed. The procedure called “3D Compare” was then run for each comparison area, using a color bar range of 0.12 mm for better color map visualization. The results report was prepared, and the “+ve average” value, which is analogous to the root mean square (RMS) value, was used as the crown fit value, which represents the cement gap.

### 2.5 Statistical analysis

The data were tabulated, and descriptive analysis was performed. Means and standard deviations (SD) were calculated for the RMS of the different areas for the marginal and internal fits. The normality of the data was evaluated using the Shapiro–Wilk test. One-way analysis of variance (ANOVA) and Tukey HSD tests were used to check for inter-group differences among the same resin material and printing orientation or the same resin and PCT. The level of significance was set at 0.05 for all tests.

## 3 Results

**Figure 3:**
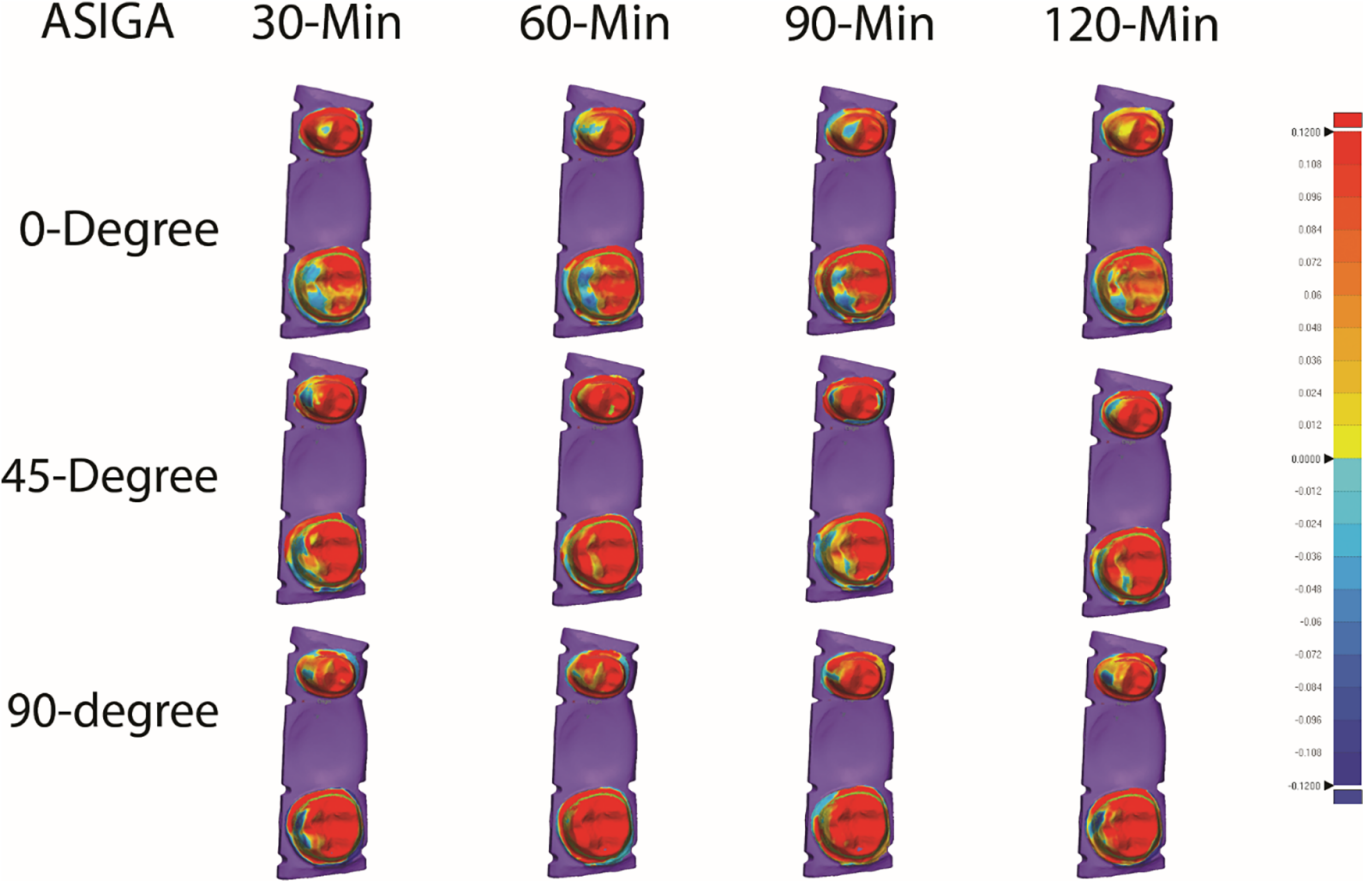
Representative color maps illustrating the internal and marginal fit of all tested ASIGA resin groups.

**Figure 4:**
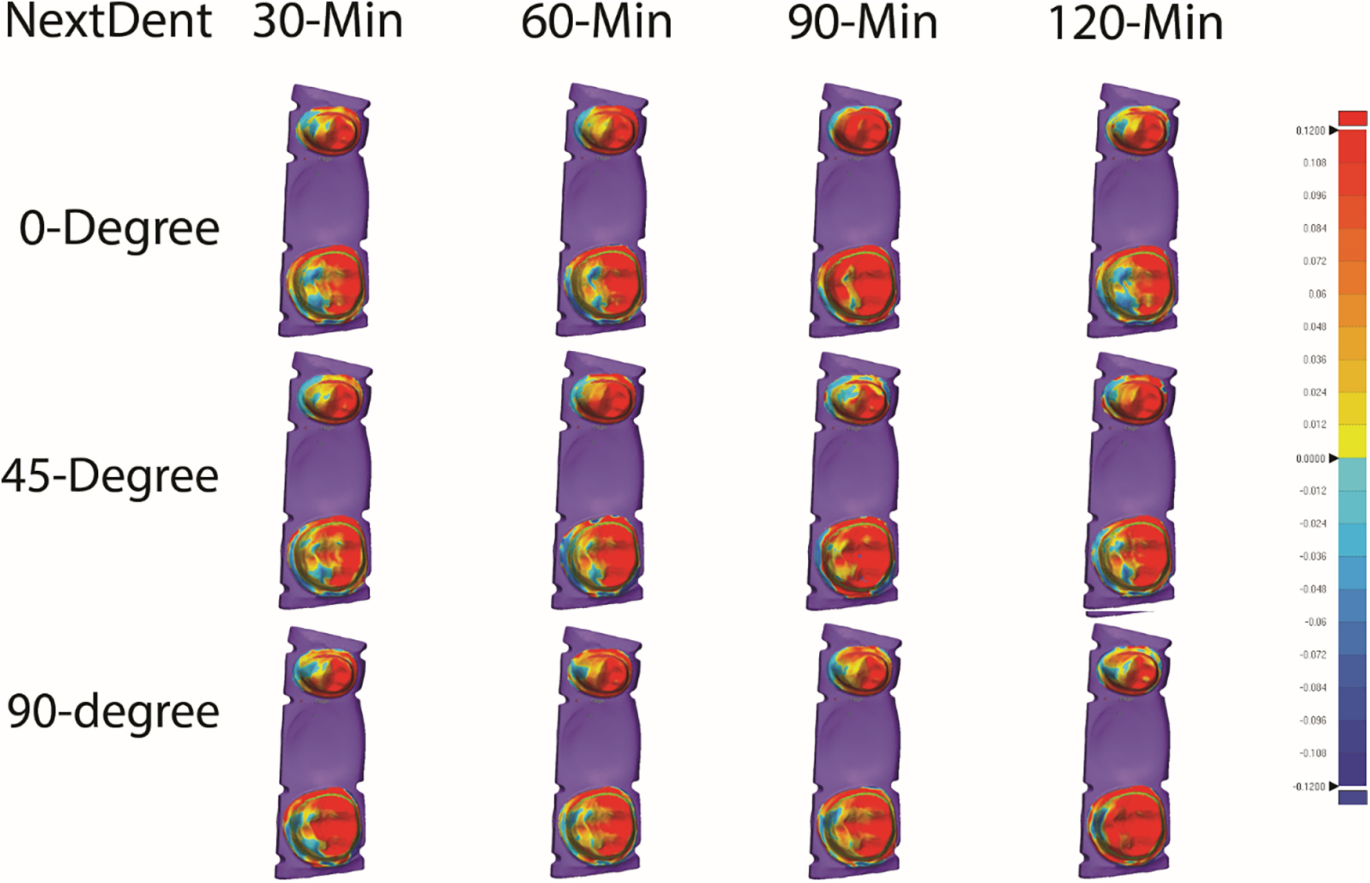
Representative color maps illustrating the internal and marginal fit of all tested nextDent resin groups.

Table 2 summarizes the mean, SD, and significance of internal fit for ASIGA and NextDent resins. ASIGA specimens printed at 45-degree showed a significant increase in the internal fit value of the premolar tooth compared with 0- and 90-degree, with no significant difference between 0- and 90-degree. Specimens with 90- and 120 min PCT, with 45-degree printing orientation, resulted in the heights RMS values (204.22 ± 67.39 µm and 205.50 ± 63.09 µm, respectively). The fit of the molar showed significant differences with different printing orientations. Specimens printed with the 0-degree group showed the lowest values, followed by 45- then 90-degree. The internal fit of the molar was generally better than the premolar when printed at 0- or 45-degree orientations. The best internal fit was detected with 0-degree/30-min PCT for the premolar and 0-degree/120-min PCT for the molar, while the worst internal fit was seen with 45-degree/120-min-PCT for the premolar and 90-degree/90-min-PCT for the molar. Regarding NextDent, no significant differences were detected between the different orientations or PCTs (*P* > 0.05) for the premolar or molar. Generally, all NextDent groups produced better internal fit than ASIGA groups (Figures 3, 4).

**Table 2:** Mean and SD of internal fit (µm) of tested groups.

Orientation	Curing time	ASIGA			NextDent		
		Premolar	Molar	Overall	Premolar	Molar	Overall
0-Degree	30 min	145.20 ± 53.50^a^	122.19 ± 21.84^a^	116.44 ± 55.59^a^	117.54 ± 14.36^a^	103.27 ± 13.39^a^	108.39 ± 12.37^a^
	60 min	147.27 ± 12.72^a^	113.75 ± 16.11^a^	118.60 ± 11.37^a^	125.21 ± 22.10^a^	118.47 ± 19.93^a^	120.94 ± 19.88^b^
	90 min	166.81 ± 66.72^a^	130.19 ± 35.02^a^	143.65 ± 46.04^b^	134.96 ± 31.15^a^	130.94 ± 30.58^a^	132.60 ± 28.40^c^
	120 min	147.53 ± 46.94^a^	108.58 ± 30.51^a^	123.94 ± 60.61^a^	116.17 ± 18.66^a^	107.17 ± 24.76^a^	110.37 ± 19.69^a^
45-Degree	30 min	178.33 ± 52.08^b^	147.11 ± 27.23^b^	157.51 ± 24.89^b^	129.05 ± 32.16^a^	119.05 ± 26.02^a^	122.83 ± 25.83^b^
	60 min	180.78 ± 81.40^b^	168.05 ± 38.56^b^	177.17 ± 48.34^c^	135.54 ± 34.15^a^	121.29 ± 18.46^a^	126.54 ± 20.38^b^
	90 min	204.22 ± 67.39^c^	151.11 ± 26.40^b^	189.83 ± 33.89^d^	116.19 ± 30.63^a^	142.04 ± 49.98^a^	133.51 ± 39.26^c^
	120 min	205.50 ± 63.09^c^	130.27 ± 18.95^a^	157.94 ± 31.14^b^	122.28 ± 27.05^a^	111.25 ± 8.27^a^	125.23 ± 12.41^b^
90-degree	30 min	158.73 ± 42.95^a^	168.94 ± 19.27^b^	158.79 ± 18.02^b^	123.28 ± 18.54^a^	115.97 ± 16.57^a^	118.59 ± 15.97^b^
	60 min	168.03 ± 60.94^a^	189.99 ± 53.59^c^	195.36 ± 53.31^d^	139.61 ± 24.51^a^	114.74 ± 22.89^a^	123.65 ± 20.18^b^
	90 min	161.01 ± 42.05^a^	199.46 ± 84.71^c^	189.51 ± 68.13^d^	114.04 ± 12.25^a^	120.85 ± 19.44^a^	118.53 ± 13.83^b^
	120 min	155.10 ± 40.17^a^	182.06 ± 62.39^c^	174.21 ± 49.21^c^	112.90 ± 26.00^a^	124.41 ± 17.92^a^	146.37 ± 28.37

Same small letter indicating in significant difference between groups per column. The significance level sat at *P* < 0.05.

For overall internal fit, for ASIGA, 0-degree at all PCTs showed the best internal fit compared to 45- and 90-degree except 0/90-min-PCT. For NextDent, 90-/120-min-PCT showed the lowest fit values compared to other groups. Also, PCT showed variations between groups within the same printing orientations, with 0/30-min-PCT and 0/120-min-PCT showing the best fit between tested groups.

Table 3 summarizes the mean, SD, and significance of marginal fit for ASIGA and NextDent resins. Regarding ASIGA, the marginal fit of the premolar was not significantly different between 0 and, 45-, and 90-degree/120-min groups. In contrast, the remaining PCT in the 90-degree orientation resulted in a significantly lower marginal discrepancy. There was no significant difference in the marginal fit of the molar with all orientations and PCTs except 0-degree/120-min, which showed the lowest marginal discrepancy. NextDent resin showed a variation in the marginal fit for the molar and premolar, where 45- and 90-degree generally produced lower marginal gaps compared to 0-degree. However, the values were not statistically significant for all PCT. Within each printing orientation, there was no significant difference in 0-degree, while 45- and 90-degree showed some differences in terms of PCTs. Among all groups, 90-degree/90-min and 90-degree/30-min-PCT produced the best marginal fit for the premolar (106.20 ± 16.73 µm) and molar (135.17 ± 22.35 µm), respectively. On the other contrary, 90-degree/120-min and 0-degree/90-min-PCT resulted in the highest marginal discrepancy for the premolar (232.17 ± 95.36 µm) and molar (243.03 ± 41.62 µm), respectively.

**Table 3:** Mean and SD of marginal fit (µm) of tested groups.

Orientation	Curing time	ASIGA			NextDent		
		Premolar	Molar	Overall	Premolar	Molar	Overall
0-Degree	30 min	191.85 ± 101.23^a^	223.76 ± 45.25^a^	258.39 ± 70.26	217.31 ± 78.06^a^	201.99 ± 32.86^a^	206.37 ± 39.47^a^
	60 min	206.79 ± 44.72^a^	217.95 ± 45.67^a^	212.11 ± 24.13^a^	212.74 ± 83.20^a^	227.17 ± 46.18^a^	223.59 ± 32.38^a^
	90 min	219.65 ± 97.52^a^	211.32 ± 66.29^a^	215.07 ± 79.11^a^	212.15 ± 71.55^a^	243.03 ± 41.62^a^	249.12 ± 42.92
	120 min	193.84 ± 71.72^a^	162.10 ± 52.24^b^	153.10 ± 61.35	212.51 ± 33.99^a^	218.42 ± 41.11^a^	216.62 ± 25.52^a^
45-Degree	30 min	207.05 ± 60.28^a^	219.78 ± 85.51^a^	217.46 ± 57.78^a^	150.46 ± 26.00^b^	161.22 ± 30.86^b^	154.88 ± 18.22^b^
	60 min	202.03 ± 91.79^a^	216.04 ± 68.27^a^	223.63 ± 61.76^a^	178.65 ± 61.91^b^	158.97 ± 39.38^b^	168.22 ± 39.58^c^
	90 min	209.88 ± 81.15^a^	221.53 ± 49.48^a^	228.68 ± 37.23^a^	107.12 ± 24.84^c^	157.59 ± 56.53^b^	137.10 ± 38.58^b^
	120 min	209.95 ± 76.13^a^	208.96 ± 39.89^a^	215.94 ± 36.62^a^	173.99 ± 44.43^b^	200.65 ± 67.17^a^	193.90 ± 37.97^d^
90-degree	30 min	174.33 ± 32.80^b^	180.67 ± 35.67^b^	178.83 ± 24.88^b^	197.50 ± 60.55^a^	170.88 ± 32.61^b^	185.14 ± 43.07^c,d^
	60 min	172.20 ± 38.34^b^	198.65 ± 35.33^a^	185.56 ± 32.57^b^	155.53 ± 45.45^b^	135.17 ± 22.35^c^	146.29 ± 33.83^b^
	90 min	174.19 ± 40.21^b^	223.13 ± 66.62^a^	201.47 ± 51.02^b^	106.20 ± 16.73^c^	136.84 ± 23.93^c^	122.17 ± 18.23
	120 min	201.39 ± 59.70^a^	229.00 ± 65.18^a^	214.48 ± 61.37^a^	232.17 ± 95.36^a^	213.95 ± 51.49^a^	224.54 ± 63.21^a^

Same small letter indicating in significant difference between groups per column. The significance level sat at *P* < 0.05.

Overall marginal fit for ASIGA results showed that 90-degree were the best among all groups except 0/120-min-PCT, which showed the lowest value (153.10 ± 61.35). At the same time, other groups of 0 and 45-degree showed high significant values except for 0/30-min-PCT, which significantly showed the highest value (258.39 ± 70.26). For NextDent, 90-degree showed the lowest value flowed by 45-degree while 0-degree showed the higher values in comparison 90-degree and 45-degree except for 90/120-min-PCT, which showed a higher significant value (224.54 ± 63.21) when compared with 0-degree.

## 4 Discussion

The accuracy of any printed object is influenced by various printing parameters, including printing technology, software selection, laser speed, laser intensity, orientation, number of layers, layer thickness, and post-processing techniques (13, 14, 32). Previous research has primarily focused on the impact of printing orientation on the quality of 3D-printed interim materials (10). Establishing the optimal orientation, a controllable parameter is crucial for achieving optimal restoration properties (19, 33, 34).

Printing orientation (PO) generally exerts a more significant influence on accuracy than PCT. A self-supported structure is essential for maintaining dimensional accuracy. Alharbi et al. (32) recommended attaching supports to surfaces with an angle less than 45-degree relative to the x-y plane. In this study, a build angle of 45 degrees was adopted, following the manufacturer's recommendations. Previous research has consistently demonstrated the influence of printing orientation on accuracy (14, 20, 32–34), with optimal results typically achieved at 135 degrees for interim crowns and 45 degrees for IFPDs (21, 34). Consequently, the methodology employed in this study adhered to these established recommendations, particularly considering the similarity between 45-degree and 135-degree printing orientations, as the same orientations were close in angle except for the support positioning in relation to the orientation (19, 35).

Previous research utilizing 3D digital superimposition has consistently demonstrated that build angles of 120 and 135 degrees, in conjunction with thin support structures, yield superior dimensional accuracy and self-supported geometry, thereby minimizing the required support surface area and reducing finishing and polishing time (20, 32). Park et al. (21) studied 3D-printed three-unit resin FPDs using five distinct build degrees (0, 30, 45, 60, and 90) and found significant variations in all groups' internal and marginal fit except the 45-degree. Osman et al. (20) similarly evaluated the 3D accuracy of DLP-printed resin crowns, and considering the combined assessment of marginal and internal fit, 45-degree was recommended as the optimal build angle. A previous study has explored methods to reduce the support area by modifying the build angle (36).

In our study, a GS was used as the control group. The rationale was to evaluate the internal and marginal fit changes between the initial uncured state (without post-curing) and the first PCT as a baseline. This helps assess post-curing's necessity by comparing the GS group's fit to those subjected to varying post-curing durations. Also, It establishes a reference point that illustrates the extent of the transformation from the initial state to a more clinically relevant and stable state and quantifies dimensional changes that occur during the post-curing process, which are critical for optimizing printing parameters for clinical applications.

While PCT has been shown to influence the accuracy of 3D-printed IFPDs (29), its impact on the marginal and internal fit of the prostheses in this study was limited. This may be attributed to the bulk of the material's already undergone polymerization, establishing its final shape and dimensions. Post-curing primarily serves to solidify the outer layer of the prosthesis. Suboptimal prosthetic accuracy can adversely influence the prognosis of restorations and increase the clinical time for insertion, adjustment, or repair (29). Numerous studies have documented shrinkage and distortion of resin during and after curing procedures (37, 38). The color maps indicate that the resin shrinkage and volumetric deformation of the 3D-printed three-unit FPDs stayed within a clinically acceptable limit of 100 µm after the post-curing process. Discussions are ongoing regarding acceptable limits for marginal gaps in 3D-printed restorations. However, previous research generally suggests that a marginal discrepancy of ≤120 µm is regarded as clinically acceptable in traditional FPDs (29, 39).

The marginal fit is the most essential factor and requirement for long-term success. The more observed marginal gaps adjacent to the pontic, as illustrated in the color maps (Figures 3, 4), are primarily attributed to polymerization shrinkage within the resins. The increased resin volume used for the pontic compared to the abutments amplified this shrinkage, leading to more significant marginal discrepancies (40). Previous research has indicated that polymerization shrinkage in resins occurs inwardly (41).

The observed marginal and internal fit variations among different build angles can be attributed to several factors. The form and surface area of the layers created by the 3D-printer differ depending on the build angle. As DLP-based 3D-printers polymerize one layer at a time, alterations in layer form inevitably lead to changes in the shape and degree of polymerization shrinkage (25, 42, 43). Jang et al. examined the marginal fit of build angles under 45 degrees and found no significant differences related to orientation. They noted that z-axis components correlated with a reduction in the marginal gap (MG), with absolute marginal discrepancy (AMD) ranging from 71.9 to 121.6 µm, consistent with prior studies. In previous research, MG values varied between 41.6 and 84.4 mm, with the 90-degree group exhibiting the highest MG at 66.1 mm (22).

The build orientation significantly influenced marginal fit, with notably lower AMD and MG values observed at 45-degree compared to 60-degree. The findings are influenced by two factors: first, printing the prosthesis at a 90-degree angle resulted in supporting structures being nearer to the lingual margin, increasing the risk of polymerization shrinkage affecting the marginal fit; second, the build orientation altered the number of polymerized layers (44). A 90-degree orientation resulted in a more significant number of polymerized layers compared to a 45-degree.

The polymerization process can compromise overall accuracy. The resin on the 3D-printer platform was subjected to light-curing, commencing with the region nearest to the platform. Given the direct connection between the supporting structures and the specimen, polymerization shrinkage within these structures could have influenced the specimen's accuracy. The amount of resin in the surrounding structures next to the edge was somewhat smaller than in the pontic, which could explain why there was no statistical significance.

The polymerization shrinkage within the pontic region substantially influenced the adjacent marginal fit. In contrast, the shrinkage of supporting structures had a minor impact on the corresponding marginal fit. The combined effect of these two factors likely contributed to the observed marginal discrepancies (2). Previous studies have corroborated the influence of supporting structures on marginal quality. Yu et al. (33) reported poor marginal quality and roughness when SLA 3D-printers were used and supports were positioned near the margins.

In conventional light-cured composite resins, the increased light intensity can lead to greater resin shrinkage and subsequent dimensional distortion, compromising marginal adaptation. Consequently, previous studies have advocated extended curing times at lower light intensities (45). However, the layer-by-layer polymerization process employed in DLP 3D printing may yield different results than traditional composite resins in post-curing shrinkage (29).

The fit and strength of provisional restorations are critical factors influencing treatment outcomes and success. This study demonstrated that printing orientation significantly impacts the fit of printed objects. While PCT had a limited effect on fit, it should be considered for optimizing strength. While beneficial for strength, prolonged PCT may negatively affect fit in certain groups. Therefore, a 0-degree orientation with 30-, 60-, or 120 min PCT is recommended for achieving optimal fit and strength with ASIGA resin material. In contrast, NextDent resin material exhibited the best-fit results with a 45-degree orientation and 30 min PCT. While variations in fit were observed among the printed groups, most exhibited clinically acceptable levels of fit.

The study highlights the clinical significance of 3D-printed provisional prostheses with key findings: Regarding material selection, NextDent resins offer a consistently superior fit to ASIGA, crucial for precise adaptation. For printing orientation, optimal orientations differ (0-degree for ASIGA, 45-degree for NextDent), aiding dental professionals in setting 3D printing protocols to improve fit and reduce chairside adjustments. Regarding PCT, personalized PCTs enhance fit differently for each resin—longer times for ASIGA and shorter for NextDent.

Regarding the limitation of this study, only two 3D-printed materials were used. One possible limitation of this study is that the cement thickness deemed suitable for the crown at all angles needed to be definitively established. Consequently, it was challenging to directly compare the differences in fit resulting from varying support positions across all build angles. Another possible limitation is the short-span FPD design used. Evaluating the internal and marginal fits of 3D-printed provisional prostheses with various lengths and designs is recommended.

Future research is warranted to investigate the influence of additional parameters, such as layer thickness, support type, and platform location, on the printing process. These factors should be carefully considered during crown fabrication. Also, investigate the effects of using different post-curing conditions and the adverse effects of over-curing. In addition to the testing fits and the materials' mechanical and optical properties after stimulating the clinical scenarios (thermal cycling, chewing simulation, and acidic challenges).

## 5 Conclusion

This study demonstrated that both printing orientation and PCT significantly influence the internal and marginal fit of 3D-printed three-unit provisional prostheses fabricated from ASIGA and NextDent resins. NextDent resin consistently outperformed ASIGA resin in terms of overall fit. Within the ASIGA group, a 0-degree orientation generally exhibited superior internal fit compared to 45- and 90-degree orientations, with some variations attributed to PCT. In contrast, the NextDent group demonstrated improved internal fit with a 45-degree orientation. Regarding marginal fit, ASIGA crowns generally exhibited better results with a 90-degree orientation, while NextDent crowns excelled with a 45-degree orientation. PCT also played a role in marginal fit, with longer durations (120 min) generally yielding better results for ASIGA resin, whereas 30 min was optimal for NextDent resin.

## Data Availability

The datasets presented in this study can be found in online repositories. The names of the repository/repositories and accession number(s) can be found in the article/Supplementary Material.
